# Seasonal Breeding and Morphological Variation Across Age and Sex in the Antioquia Brushfinch (*Atlapetes blancae*)

**DOI:** 10.1002/ece3.72408

**Published:** 2025-11-12

**Authors:** Juan Pablo Gomez, Sergio Chaparro‐Herrera

**Affiliations:** ^1^ Departamento de Química y Biología Universidad del Norte Barranquilla Colombia; ^2^ Proyecto Atlapetes: Ecología y Conservación del Gorrión‐Montés Paisa (Atlapetes blancae) Medellín Colombia; ^3^ Grupo de Ecología y Evolución de Vertebrados Universidad de Antioquia Medellín Colombia

**Keywords:** Colombia, endangered endemic species, environmental determinants of reproduction, HOF models, morphometrics, timing of reproduction

## Abstract

Describing the natural history of endemic and endangered species is useful for developing effective conservation plans. Two important pieces of the natural history are the timing of reproduction and morphometrics. In this study, we estimated the reproductive season of Antioquia Brushfinch ( *Atlapetes blancae* ) based on a large database of observations and captures. We also provide the most extensive morphometric data base of males, females and subadult individuals for this species. Taking advantage of ecological modeling techniques, we estimated that the reproductive season of *A. blancae* most likely spans from February through late July with peak reproductive activity during early July. The reproductive activity is influenced by day length and precipitation. We also found that subadults are smaller than adults and females are smaller than males providing evidence for both, age and sexual size dimorphism. This study was the first attempt to provide important missing information about *A. blancae* that could help with the understanding of population viability.

## Graphical abstract

The reproductive season of *A. blancae* most likely spans from February through August with peak reproductive activity during May, and is highly correlated to day length, and precipitation during the first rainy season of the year, furthermore, it is also highly correlated with the reproductive activity of many other species in the genus *Atlapetes*. Additionally, juveniles are smaller than adults and females are smaller than males providing evidence for both, age and sex size dimorphism. With this study, we now can focus conservation actions on periods of reproduction which are important bottlenecks of bird populations.

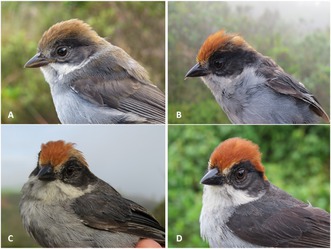

## 1 Introduction

Reproduction is a key event in the life cycle of organisms, sustaining both individual fitness and population persistence. It is regulated by ultimate and proximate factors such as climate, resource availability, day length, competition, and predation (Wolf 1969; Echeverry‐Galvis and Córdoba‐Córdoba 2008). Understanding reproductive patterns therefore informs not only evolutionary processes (Wiersma et al. 2007; Jetz et al. 2008; Covas 2012; Pienaar et al. 2013), but also the demographic dynamics of endangered species (Krabbe 2004; Krabbe et al. 2011; Van Allen et al. 2012; Rodríguez‐Linares et al. 2019).

In temperate regions, avian reproduction is typically unimodal, constrained primarily by photoperiod, resource availability, and summer climatic conditions (Lack 1968; Dawson et al. 2001). In tropical regions, many species also reproduce seasonally, often in synchrony with rainfall or photoperiod (Poulin et al. 1992; Wikelski et al. 2000), although in some taxa reproductive activity peaks following the rainy season (Araujo et al. 2017). In both tropical and temperate birds, the onset of reproduction is largely regulated by increasing day length (Hau et al. 1998; Dawson et al. 2001). Such synchrony in breeding phenology among tropical birds is frequently associated with increases in insect biomass, a key resource for nestling development (Hau et al. 2000). Other species, however, breed opportunistically or throughout the year, exhibiting no distinct reproductive peak (Stouffer et al. 2013). Alterations in resource dynamics under changing climatic conditions may generate phenological mismatches, with potential consequences for population stability and persistence (Belitz et al. 2025). Understanding whether species exhibit reproductive synchrony, and the environmental cues that govern its timing, is essential for elucidating life‐history strategies and informing conservation planning (Goymann and Helm 2014; Moreno‐Palacios et al. 2025). Land managers, for instance, often schedule habitat management actions (e.g., mowing, prescribed burning, logging) outside of the breeding season to mitigate impacts on reproductive success and population size (Kubacka et al. 2014; Antoniazza et al. 2018). Similarly, restoration initiatives increasingly integrate phenological information to align resource availability with the breeding requirements of focal species (Slankard et al. 2024).

Beyond phenology, morphological differences across sexes and life stages provide insights into ecological and evolutionary processes (Owens and Hartley 1998; Tellería et al. 2013; Gonzalez‐Voyer et al. 2022). For instance, male‐biased sexual size dimorphism is often attributed to sexual selection through male–male competition, whereas female‐biased dimorphism can reflect fecundity advantages (Darwin 1874; Székely et al. 2000). Although male‐biased dimorphism is more widespread, evidence also supports the fecundity hypothesis in many taxa (Caron and Pie 2024). Similarly, differences in morphology between juveniles and adults facilitate age classification in the field (Sanchez et al. 2021), which is critical for population monitoring and demographic modeling.

The Antioquia Brushfinch ( *Atlapetes blancae* ) is a Critically Endangered species endemic to Colombia, threatened by habitat loss and a presumed small and declining population (Renjifo et al. 2014; BirdLife International 2021; Chaparro‐Herrera et al. 2024). Rediscovered in 2018, this species has since been the focus of monitoring efforts aimed at documenting its ecology and natural history (Correa et al. 2019; Chaparro‐Herrera and Lopera‐Salazar 2019; Valencia et al. 2019; Chaparro‐Herrera et al. 2021, 2024; Díaz‐Vallejo et al. 2023; Velázquez‐Tabares et al. 2025). Despite this recent progress, key aspects of its biology remain unknown, including its reproductive phenology, environmental cues for breeding, and morphological differentiation across sexes and age classes. Previous reports are limited to isolated descriptions of subadult and reproductive individuals (Correa et al. 2019) and nest characteristics (Chaparro‐Herrera and Lopera‐Salazar 2019). This lack of information is not unique to *A. blancae* : reproductive and morphological data are scarce across the genus *Atlapetes* and more broadly among Neotropical birds (Echeverry‐Galvis and Córdoba‐Córdoba 2008; Xiao et al. 2017; Lees et al. 2020; Billerman et al. 2025).

Available evidence suggests that most species in the genus *Atlapetes* breed during a unimodal season, primarily in the first half of the year, with reproductive activity concentrated from March to July but extending in some cases until September (Rowley 1962; Hilty and Brown 1986; Stiles and Skutch 1989; Fjeldså and Krabbe 1990; Oppel et al. 2003; Cisneros‐Heredia 2006; Greeney and Nunnery 2006; Biancucci and Martin 2008; Olaciregui and Botero‐Delgadillo 2012; Utria 2012; Billerman et al. 2025). Fewer species breed during the second half of the year (typically October–November), sometimes extending to March (Willard et al. 1991; Barrowclough et al. 1995; Salaman et al. 1998; Capllonch et al. 2014; Forrester and Londoño 2016; Billerman et al. 2025). Only three species have been reported to breed year‐round (Hilty and Brown 1986; Fjeldså and Krabbe 1990; Cisneros‐Heredia 2006; Janni et al. 2008; Greeney 2009; Peraza 2009; Greeney et al. 2010, 2011; Billerman et al. 2025). Nevertheless, breeding information remains unavailable for more than 30% of species in the genus, including *A. blancae* . Recent work in a closely related species suggests that breeding may be linked to rainfall in preceding months (Castaño et al. 2023), but potential environmental drivers have not been evaluated across the genus. Similarly, sexual size dimorphism has not been documented in any *Atlapetes* species, and quantitative morphological data for juveniles are lacking.

Here, we combine 6 years of field observations and morphological data to provide information about the phenology and morphology of this species. Our objectives were to: (1) estimate the reproductive phenology of *A. blancae* and evaluate the influence of abiotic factors, (2) compile the most comprehensive morphological dataset for this species to date, and (3) quantify morphological variation across life stages and sexes. Based on evidence from related taxa, we hypothesized that *A. blancae* exhibits a unimodal breeding season coinciding with the onset of the rainy season, likely triggered by increased fruit and insect availability that peaks following the first rains after the prolonged dry season. However, given the bimodal rainfall pattern across its distribution (March and August peaks), we also considered the possibility of year‐round reproduction. Finally, based on the absence of reported sexual dimorphism in the genus, we expected no significant morphological differences between males and females since it has been reported that this trait exhibits strong phylogenetic signal (Caron and Pie 2024). Nonetheless, there is substantial variation in the presence of sexual size dimorphism in passerines which supports the importance of still exploring the possibility for such a pattern (Owens and Hartley 1998).

## 2 Methods

### 2.1 Study Area

**FIGURE 1:**
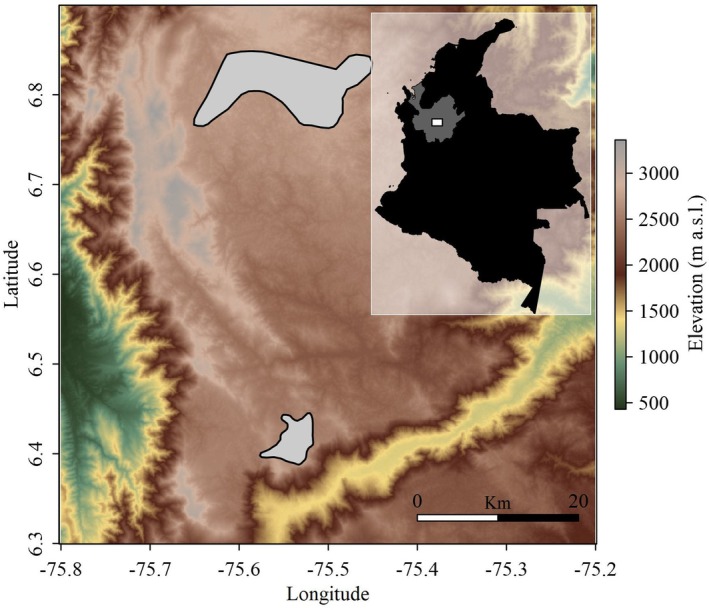
Map showing the location of the study area where evidence for reproductive activity was collected. Polygons in gray in the main panel show the areas within the Altiplano of Santa Rosa de Osos where individuals were captured and observations of other reproductive activities were collected. This map has been modified from Chaparro‐Herrera et al. (2024).

To study the reproductive activity and morphology of *Atlapetes blancae* , we obtained records from montane scrub or montane shrublands, *A. blancae's* preferred habitat (see Chaparro‐Herrera et al. 2021), in the Altiplano de Santa Rosa de Osos (ASRO), municipalities of San Pedro de los Milagros (San Pedro from here on) and the locality of Aragon at the municipality of Santa Rosa de Osos (Aragon from here on), in the Antioquia department, northern Central Andes of Colombia, between 2493 and 2837 m above sea level (Figure 1).

### 2.2 Data Collection

**FIGURE 2:**
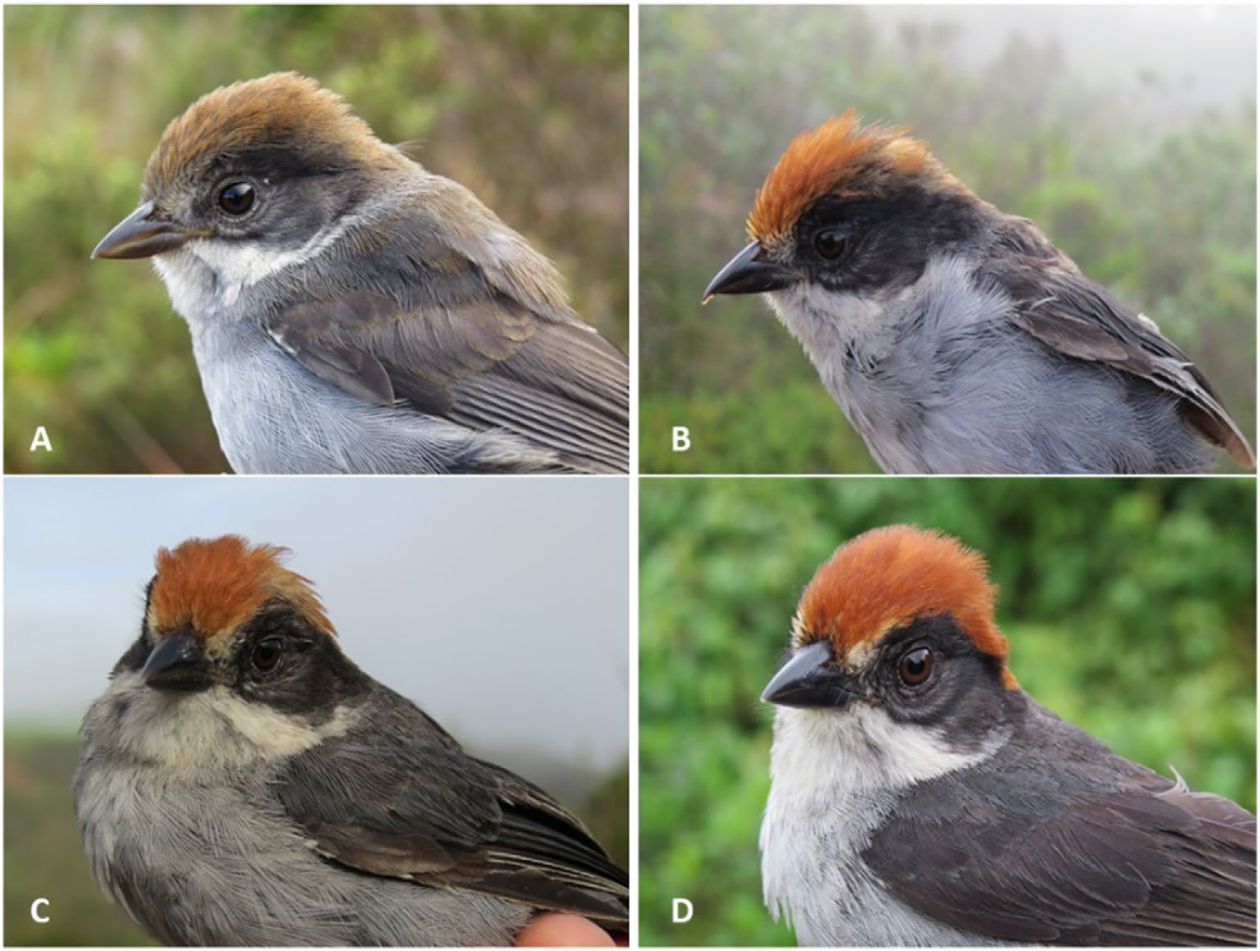
Pictures of (A) juvenile; (B, C) subadult, and (D) adult individuals of Antioquia Brushfinch ( *Atlapetes blancae* ).

To obtain information about *Atlapetes blancae* 's reproductive activity, we first performed a literature search of all published databases, observations and captures beginning in January 2018 through November 2023. In this search we included peer‐reviewed reports (Correa et al. 2019; Chaparro‐Herrera and Lopera‐Salazar 2019; Valencia et al. 2019; Chaparro‐Herrera et al. 2021), and observations reported to eBird (2023; reported until November 2023) with pictures of subadult and juvenile individuals. Second, we constructed a database with unpublished records collected by researchers in our group during the development of different projects (Proyecto Atlapetes; https://arcg.is/1yz0eK). The database consisted of georeferenced and dated records with information about the age of the individuals recorded (i.e., subadult, juvenile and adult) and included reports of nesting activity. To objectively differentiate adult, juvenile and subadult individuals, we classified juvenile individuals as having a pale gape, a blackish‐yellow mandible, a dark brown crown, a grayish‐black mask, a brown back with grayish tints, a whitish‐gray chest with slight black striations (grayish or absent when approaching its subadult stage) and a whitish‐brown belly (Figure 2A). Subadult individuals' plumage is similar in structure, patterns and tonal coloration to adults, but do not have the definitive adult plumage (Figure 2B,C). Instead, plumage is in general opaquer and can be seen as an intermediate stage between juveniles and adults (Figure 2D). Unfortunately, the time that individuals retain juvenile or subadult plumage is unknown. The only information we have based on gape coloration, and our observations is that juvenile individuals are younger than subadults. We speculate that juvenile individuals retain this coloration during the first months when they shift to subadult plumage retaining it for a few months more. Although subadult passerines may retain this plumage for a full year (Hawkins et al. 2012), we believe that this is not the case since we do not have evidence of the presence of subadult individuals year‐round.

In addition, we pooled the observational database with information based on individuals captured in the field (55 individuals). During May and July 2022, as part of a mark‐recapture project to estimate population size and monitoring program of *A. blancae* (Chaparro‐Herrera et al. 2024), we visited seven localities in our study area to capture, measure, sex, and band the largest number of individuals possible (see Chaparro‐Herrera et al. 2024 for sex determination). In total we spent 18 full days in these localities (between 3 and 6 days per locality) capturing unmarked focal individuals using mist‐nets. We obtained seven morphological measurements: weight, total culmen, culmen taken from the nares, gape, bill height and width at the nares, tarsus, wing, tail, and total length. These are standard morphological measurements that are usually available for birds and have been linked to important ecological processes (Tobias et al. 2022). We inspected evidence of molting and general plumage status and evidence of reproduction based on incubation patch and cloacal protuberance (Ralph et al. 1996).

## 3 Data Analysis

### 3.1 Breeding Phenology

To estimate the phenology of *A. blancae* we used the compiled database with evidence of reproductive behavior and the exact date at which the evidence was collected. The dates were then assigned to one of the 52 weeks in the year. Using the presence or absence of evidence for reproduction every week in a year, we estimated the breeding phenology for *A. blancae* using binomial hierarchical logistic regressions in the form of Hulsen‐Olff‐Fresco (HF) models using the eHOF package in R (Jansen and Oksanen 2013; R Core Team 2022). An observation of a juvenile, a subadult, an active nest a female with a brood patch or a male with cloacal protuberance would constitute evidence of reproduction and be coded as 1. In contrast, observations of adults in breeding plumage with none of these signs would constitute a lack of evidence of reproduction for a given week and be coded as 0. The data base showed that there was a gap between weeks 31 and 47 for which we found no reproductive evidence. Thus, we investigated different starting dates for reproduction by comparing the fit of the models starting in the first week of the year with models starting in week 47. Although HOF models are usually used to model the distribution of species along environmental gradients, we consider that this type of model is also appropriate to estimate the reproductive season of a species across a year. The reproductive season, as species distribution may be constant across time or have hump‐shaped forms that may be symmetric or asymmetric and may be unimodal or multimodal. Similar approaches have been used to identify constant, unimodal or multimodal reproductive phenology in other organisms (Durant et al. 2013; Willig and Presley 2023). To model *A. blancae* 's phenology, we evaluated the fit of seven models: (I) a constant, (II) monotonic, (III), stepwise, (IV) symmetric double logistic, (V) asymmetric double logistic and models (VI), and (VII) which are modifications of IV and V to include bimodal responses (Jansen and Oksanen 2013). These models allow for many possible shapes of the reproductive season across a single year. Model selection was performed based on the Bayesian Information Criterion (BIC) and we assumed that there was strong evidence in favor of the model with the lowest BIC when differences in BIC between models were larger than three units. However, it has been recognized that sometimes the logistic model is unstable and parameters are not identifiable (Jansen and Oksanen 2013). Thus, we used a second model selection approach in which we used 500 replicates of non‐parametric bootstrap and for each bootstrapped data set we performed model selection. We then selected the model that was deemed as best the largest number of times. Finally, we estimated the core of the reproductive season as the central border and the peak of the reproductive season as the optimum value from the logistic fit (see Heegaard 2002 for definitions of central border and optimum value). Because data from captures were restricted to the period from May to June, to avoid biases in the estimation of the reproductive season because of limited data, we performed three analyses. The first one used the entire dataset that included all types of evidence, a second analysis included only evidence from the presence of subadult and juvenile individuals and a third analysis included only the presence/absence of incubation patch, cloacal protuberance or active nests.

We accounted for potential biases in the estimation of reproductive behavior due to sampling effort; we compiled a database with individual observations throughout the year. In this case we did not discriminate between adults, juveniles and subadult individuals. For this, we summed the total number of individuals observed during a week and used a linear model between logit transformed estimated probability of reproduction and the number of individuals recorded for that same week. We then compared the BIC of the latter model with an intercept‐only model. A significant effect of individuals on the probability of reproduction would indicate a sample size bias in our analysis.

### 3.2 Effect of Environmental Variables on Reproduction in *Atlapetes blancae*

To evaluate the potential influence of precipitation and day length on the probability of reproduction, we fitted generalized least squares (GLS) models with logit‐transformed predicted probabilities of reproduction as the response variable, and day length and precipitation as predictors, including the interaction between the independent variables. Since day length and precipitation were highly collinear (*R* > 0.7), we used as an independent variable for the models, the residuals of the relationship between day length and precipitation. This allowed us to evaluate the effect of precipitation alone after the variance explained by day length was excluded. Precipitation and daylength were also centered before analysis by taking the variable's mean from each observation. Scaling allowed us to directly compare the magnitude of the effect of the independent variables on the probability of reproduction. To account for temporal autocorrelation, we specified a first‐order autoregressive correlation structure. We compared all possible model combinations of day length and precipitation using the Bayesian Information Criterion (BIC). The model with the lowest BIC was considered the best supported, and we assumed strong evidence in its favor when the difference in BIC between competing models exceeded three units. Analyses were conducted in R (R Core Team 2022). Day length (hours of sunlight) for the first day of each calendar week was extracted using the daylength function from the *geosphere* package (Hijmans 2021). Weekly precipitation data were obtained from the Colombian Institute of Hydrology, Meteorology and Environmental Studies (IDEAM, http://dhime.ideam.gov.co/atencionciudadano/) by searching for active weather stations in the municipalities of Santa Rosa de Osos and San Pedro de los Milagros with daily precipitation records from 1980 to 2024. Only stations 27,010,840 and 27,010,880 met these criteria. Daily precipitation data were aggregated into weekly totals by summing rainfall across seven‐day periods. We then calculated mean weekly precipitation across all years with available data. Missing daily values were imputed using the mean precipitation from the three preceding and three subsequent days.

### 3.3 Morphological Differences

To evaluate morphological differences among individuals in different classes, we first used a principal components analysis (PCA) to reduce the number of morphological variables into an uncorrelated set of composite variables. To account for allometry in morphological traits, the PCA was performed using the log transformed values of the traits (Klingenberg 2016). The first principal component (PC) in this analysis is usually related to size, and the rest of the PCs are related to shape (Klingenberg 2016). We retained the principal components that explained more than 10% of the variation in the original data and had an eigenvalue larger than 1 (Legendre and Legendre 1998). Second, to test for differences in size among individuals in different sexes and life stages, we used an analysis of variance (ANOVA) with sex and life stage as explanatory factors and the interaction between the two factors. Third to test for differences in the shape of traits accounting for allometry, we performed a multivariate ANOVA (MANOVA) with the retained PCs, excluding the first one, as response variables and sex and life stage as independent variables. Finally, we also ran separate analyses of variance for age groups and sex for raw variables and reported the Bonferroni corrected *p* values; these analyses were performed in R (R Core Team 2022).

## 4 Results

### 4.1 Breeding Season

**FIGURE 3:**
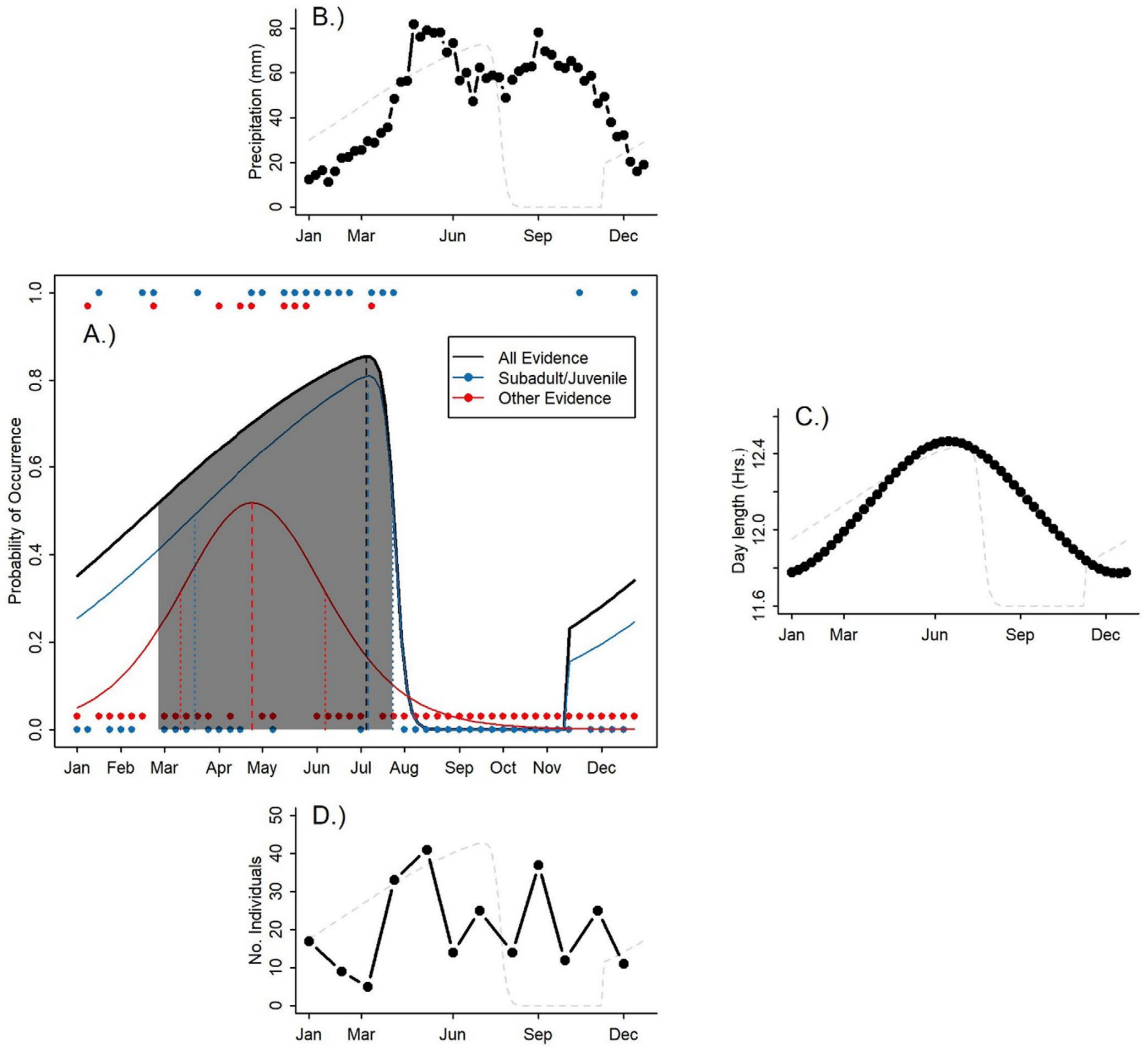
Estimated breeding phenology of Antioquia Brushfinch ( *Atlapetes blancae* ) based on presence/absence data of reproduction from different sources. (A) We show the weekly probability of reproductive activity along the year based on all evidence (black line), presence/absence of subadult and juvenile individuals only (blue line) presence/absence of incubation patch, cloacal protuberance or active nests (red line). The shaded polygon shows the estimated core, and dashed lines show the peak of the reproductive season based on all evidence together. Similarly, dashed and dotted blue and red lines show the peak and core estimated reproductive activity based on the presence of subadult/juvenile individuals or other evidence, respectively. We also show important environmental variables (B) monthly precipitation and (C) daylength across the year that may determine the reproductive activity and (D) Shows the number of *A. blancae* individuals from which we obtained information. In panels (B–D), the gray dotted line shows the prediction of the probability of observing reproductive activity along the year as reference for comparison precipitation, daylength and sampling effort.

Our database consisted of 106 records with evidence of reproduction between nesting (12), subadult (27), juvenile (39), incubation patch (11), and cloacal protuberance (17) throughout the year. For both, the complete data set and the dataset including only subadults and juveniles, the best model was model V with the starting date on the second week of November. This model corresponds to a unimodal asymmetric double logistic model. Using the full data set, model V had the lowest BIC with the second‐best model being model III with a difference in BIC of 2.5 units (Table 1). Furthermore, model V was selected as best in 60% of the bootstrap replicates. The total data set suggested that *A. blancae* 's breeding season spans from week 7 through week 30 and with the highest reproduction activity predicted at week 27 (Figure 3A). This means that the reproductive season spans from late February through late July with a peak in mid‐July (Figure 3A). Using data with juveniles and subadults only yielded almost identical results as the model using the entire data set (Table 1; Figure 3A). In this case, the reproductive season was predicted to peak in week 28 with a central reproductive season between weeks 12 and 30 (Figure 3A). Finally, using the data from nesting records, presence of cloacal protuberance and incubation patch, the best model selected was the unimodal model IV with a peak in reproductive activity in week 17 and the core happening between week 10 and 23 (Figure 3A). Regarding sampling effort (days/month), we compiled a database with 231 records during all months of the year. May was the month with the greatest sampling effort with 41 observations, followed by September and April with 37 and 33 observations respectively. The months with the smallest sampling effort were March and December with five and three records (Figure 3D). The number of observations was not related to the probability of reproduction since the model including sampling size had a BIC 3.4 units larger than the null model with intercept only (Linear Model; −3.8 (95% CI = −7.1 to −0.5)–0.17 (95% CI = −0.6 to 0.3)*Samp.Size; BIC = 386.8, BIC null = 383.4, *R* ^2^ = 0.01, Figure 3D).

**TABLE 1:** Summary statistics for models estimating the reproductive phenology of *Atlapetes blancae* .

Model	Start week	*N*	Npar	All				Juv				Other evidence			
				Lik	BIC	dBIC	Bs	Lik	BIC	dBIC	Bs	Lik	BIC	dBIC	Bs
V	47	52	4	−21.8	59.4	0.0	60	−21.9	59.5	0.0	52				
III	47	52	3	−25.0	61.9	2.5	22	−25.5	62.8	3.2	12				
VII	47	52	5	−22.8	65.4	6.0	12	−21.0	61.7	2.1	28				
VI	1	52	4	−26.7	69.1	9.7	30	−25.9	67.6	8.1	31	−17.9	51.6	4.0	3
VII	1	52	5	−25.3	70.3	11.0	23	−25.7	71.2	11.7	34	−16.5	52.8	5.1	8
IV	47	52	4	−27.3	70.3	11.0	3	−27.7	71.2	11.7	2				
VI	47	52	4	−27.3	70.3	11.0	2	−27.7	71.2	11.7	4				
IV	1	52	3	−29.8	71.5	12.1	16	−29.4	70.6	11.1	11	−17.9	47.7	0.0	40
II	1	52	2	−32.8	73.4	14.1	11	−32.7	73.4	13.9	1	−21.4	50.6	2.9	8
I	1	52	1	−35.1	74.1	14.7	8	−33.5	71.0	11.5	16	−24.0	51.9	4.2	5
I	47	52	1	−35.1	74.1	14.7	0	−33.5	71.0	11.5	0				
II	47	52	2	−33.6	75.1	15.7	0	−32.8	73.6	14.1	0				
III	1	52	3	−31.7	75.2	15.8	5	−31.9	75.6	16.1	0	−17.9	47.7	0.0	23
V	1	52	4	−29.8	75.4	16.1	7	−29.3	74.5	15.0	7	−17.2	50.2	2.5	13

*Note:* Models are ordered by Bayesian Information Criterion (BIC), from smallest to largest. **All** refers to the model using all available data; **Juv** refers to the model using only juvenile data; and **Other Evidence** models are based on data from cloacal protuberances, incubation patches, or the presence of nests. *Start Week* = week of the year used as the initial week for model estimation; *N* = sample size; *Npar* = number of model parameters; *Lik* = model log‐likelihood; *BIC* = Bayesian Information Criterion; *dBIC* = difference in BIC relative to the best‐supported model; *Bs* = proportion of bootstrap replicates in which the model was selected as best.

### 4.2 Effect of Precipitation and Daylength on Reproductive Activity

We found that the probability of observing reproductive activity in *A. blancae* was increased with increasing daylength and precipitation and the interaction between daylength and precipitation (Figure 3). We found that the model with the lowest BIC was the model including daylength, precipitation and the interaction between these two variables (Table 2; BIC = −300.6). The second‐best model was the null with 4.8 BIC units more than the best model (Table 2).

**TABLE 2:** Summary statistics for models relating precipitation and daylength with weekly probability of reproduction.

Model	Intercept	DayLen	Prec	DayLen*Prec	df	logLik	BIC	dBIC
~DayLen*Prec	−4.52	15.68	−0.33	1.15	6	−139.25	302.2	0.00
~1	−3.73				3	−147.58	307.0	4.82
~Prec	−4.24	−0.16			4	−145.68	307.2	4.96
~DayLen+Prec	−3.88	8.56	−0.18		5	−145.32	310.4	8.20
~DayLen	−3.26	7.12			4	−147.42	310.6	8.44

*Note:* The models account for temporal autocorrelation using an autoregressive structure of first order (i.e., ARMA(1)).

### 4.3 Morphological Differences Among Age Groups and Between Sexes

We captured 55 individuals of *A. blancae* , from which we obtained morphological traits and sex. From the 55 individuals, 11 were captured in San Pedro (5 males, 6 females) and 44 in Aragon (20 males, 24 females), of which 37 were adults (67.27%, 21 males, 16 females). One adult individual was excluded from posterior morphological analysis because it was missing its rectrices at the time of capture. Six of the individuals captured were juveniles (10.9%, 1 male, 5 females) and 12 were subadults (21.8%, 3 males, 9 females). Of the adults, seven individuals showed incubation patches and 16 cloacal protrusions (see Supporting Information S3 for details of individuals' capture dates and locations). All individuals were sexed molecularly; sexing methods are presented elsewhere (Chaparro‐Herrera et al. 2024).

**FIGURE 4:**
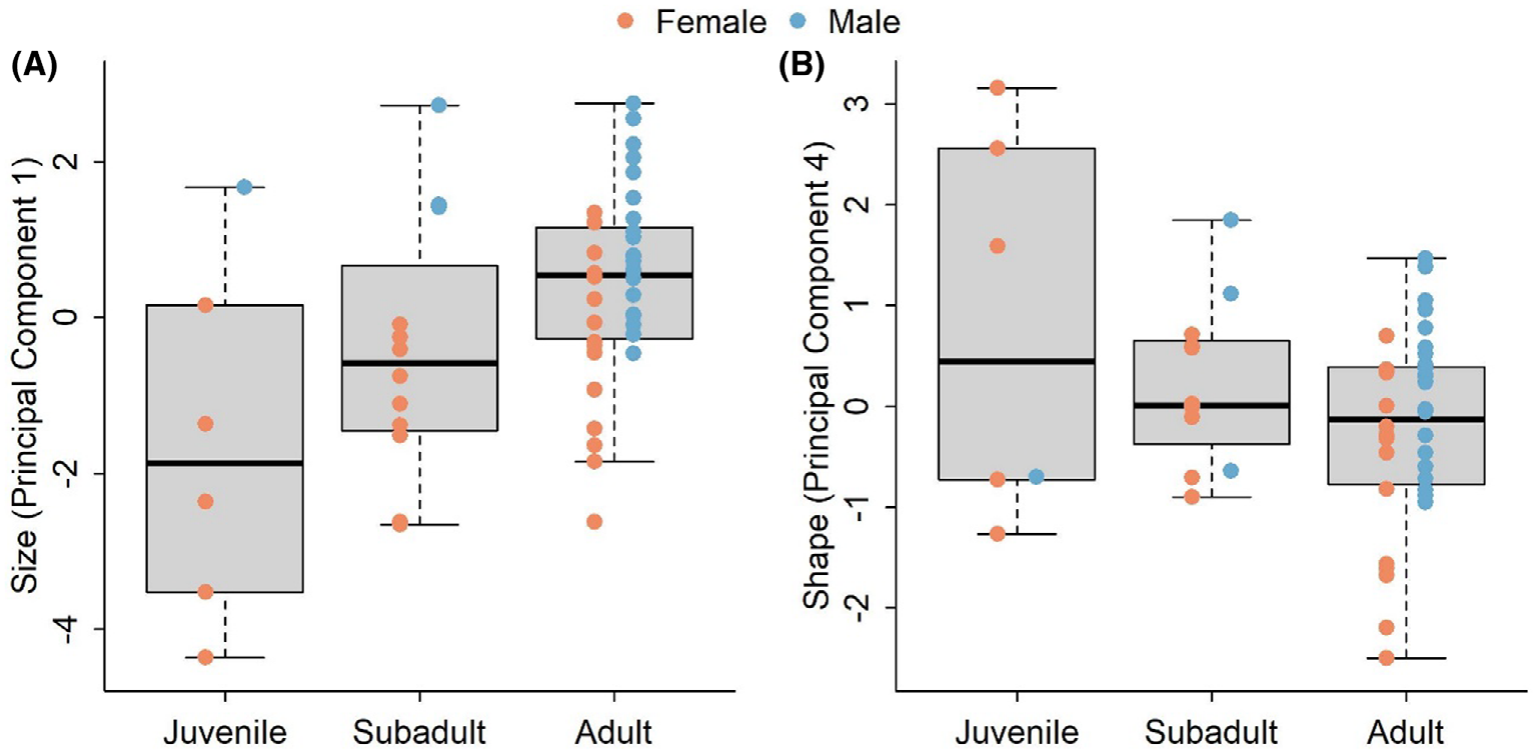
Boxplots showing morphological differences between adult, juvenile and subadult individuals in (A) Size and (B) the fourth principal component related to shape of morphological traits in Antioquia Brushfinch ( *Atlapetes blancae* ). Size and Shape were obtained from a principal component analysis based the log transformed values of raw trait values. The first principal component analysis was related to size, and the rest were related to shape. We show only the principal component that had marginal significant differences among sexes and age groups.

Principal component analyses resulted in four reduced variables that explained 63% of the variability. In general, PC1 was related to size, PC2 to bill and tarsus length, PC3 to bill height and width and hallux length and PC4 to gape (Figure S1; Table S1). The analyses of variance showed significant differences between juveniles and adults and between males and females in size but not in shape (Figure 4; Table 3). Bonferroni corrected analyses of variances on raw variables showed significant differences between adults and juveniles on gape, tail length and total length and marginally on wing length (Figure S2; Table 3). Additionally, males and females were significantly different on bill height, exposed culmen and wing, tail and total length and marginally on weight (Figure S2; Table 3). The shape analysis suggested that there was no significant difference in shape among life stages or among sexes (Table 3). The results of the Manova, however, when exploring the univariate responses showed marginal differences among life stages in PC4 (Figure 4).

**TABLE 3:** Results from the analyses of variance comparing morphological variables of Antioquia Brushfinch ( *Atlapetes blancae* ), among age groups and among sexes and the interaction (Age × Sex) between age group and sex based on principal components and on raw variables.

	*N*	Age groups		Sex		Age × Sex	
		*F*	*p*	*F*	*p*	*F*	*p*
*Principal components*							
Size	54	3.35	**0.04**	45.99	**< 0.001**	4.12	**0.02**
Shape	54	1.51	0.16	0.6	0.66	1.8	0.08
PC2	54	0.39	0.68	0.31	0.58	1.04	0.36
PC3	54	1.34	0.27	0.06	0.8	0.64	0.53
PC4	54	4.6	**0.01**	2.05	0.16	2.8	0.07

	*N*	Age groups		Sex		Age × Sex		PC
		*F*	*p*	*F*	*p*	*F*	*p*	
*Raw variables*								
Weight	54	0.7	0.5	2.76	0.1	0.59	0.55	1
Bill height	54	0.34	0.71	4.09	0.05	0.65	0.53	3
Bill width	54	0.78	0.46	0.22	0.65	0.22	0.8	3
Gape	54	8.1	**0.001**	0.88	0.35	2.86	0.06	4
Total Culmen	54	0.57	0.57	0.02	0.88	2.41	0.1	2
Exposed Culmen	54	0.19	0.82	8.72	0.004	2.14	0.12	2
Tarsus	54	1.05	0.356	0.15	0.7	0.78	0.46	2
Hallux	54	1.07	0.351	0.78	0.38	1.2	0.31	3
Wing length	54	7.77	**0.001**	80.432	**< 0.001**	1.88	0.16	1
Tail length	54	4.93	**0.01**	3.38	0.07	5.27	**0.008**	1
Total length	54	5.15	**0.01**	4.85	**0.03**	1.35	0.26	1

*Note:* Size corresponds to the first principal component and Shape refers to summary statistics of the Multivariate Anova. Lines labeled as PC2, PC3 and PC4 are the univariate response of the principal components to each factor and its interaction. The column labeled as PC shows the principal component with which each variable was most related. The relationship between the raw variable and the PC was determined using the largest absolute value of the loadings. Bold values denote statistical significance at the *p* < 0.05.

## 5 Discussion

Until now, little information has been available on the reproductive biology and phenology of *A. blancae* . Chaparro‐Herrera and Lopera‐Salazar (2019) provided the first description of a nest and eggs, reported some reproductive behaviors, and suggested that the breeding season likely occurs between January and May. Here, we provide more robust evidence of reproductive phenology for this species based on 6 years of observations, showing that the breeding period is considerably longer. Our data indicate that *A. blancae* begins breeding as early as late December, with a core reproductive season spanning from late February through early August. This season appears to be influenced by both day length and precipitation (Figure 3; Table 2). The probability of reproduction increased with increasing daylength and precipitation after the longest dry season. In addition, we present the most comprehensive morphological dataset available for this species, demonstrating significant size differences between sexes and age classes, with adults and males being larger than subadults, juveniles, and females (Figure 4).

### 5.1 Reproductive Phenology

The timing of reproduction in birds can be mediated by environmental factors such as precipitation, temperature, and day length (photoperiod). Photoperiod has been especially well documented in temperate zones, where strong seasonal variation exerts a marked influence on breeding activity (Wingfield et al. 1992; Hau et al. 1998, 2008; Dawson et al. 2001; Sharp 2005; Boyle et al. 2020; Castaño et al. 2023). In tropical regions, however, precipitation seasonality is generally the dominant driver, insect and fruit biomass peak during the rainy season providing additional resources for reproduction (Wolda 1978; Martin et al. 2000; Wikelski et al. 2000; Ahumada 2001; Boyle et al. 2020; Castaño et al. 2023). Our results support the resource abundance hypothesis, since we found that the breeding season of *A. blancae* is closely associated with precipitation patterns across its distribution (i.e., reproductive activity peaks during the rainy season). This is consistent with other Colombian Andean birds, many of which show breeding peaks between March and June that coincide with rainfall (Moreno‐Palacios et al. 2025), including closely related species in the Passerellidae family (Castaño et al. 2023).

Interestingly, despite similar rainfall amounts during the second half of the year, *A. blancae* shows little or no breeding activity (Figure 3B). This suggests that both physiological and energetic constraints, beyond resource availability, may limit reproduction. Given that the species inhabits areas with relatively high annual precipitation (< 2000 mm), resource scarcity alone is unlikely. Our observations indicate that *A. blancae* relies heavily on insects and berries from shrubs in the genera *Vaccinium*, *Melastomataceae*, and *Ericaceae* (Chaparro‐Herrera et al. 2021). It remains unclear whether the diet shifts seasonally—for example, from protein‐rich insects to fruit—depending on the breeding stage. Such seasonal shifts have been documented in highly frugivorous tropical birds (Riehl and Adelson 2008). One potential explanation is that insect pulses during the first rainy season provide excess protein critical for reproduction, as documented in other highly seasonal ecosystems (Silva et al. 2011). Additionally, the fact that different bird guilds in Colombia breed in different seasons suggests that species may track resources according to their specific requirements (Moreno‐Palacios et al. 2025).

Physiological factors may also play a role. Tropical birds tend to have lower metabolic rates, which affect their pace of life and investment in reproduction (Wikelski et al. 2003; Martin 2004). Molt, for example, could impose energetic constraints during parts of the year when resources are otherwise sufficient. However, the timing of reproduction is highly variable in tropical birds, and thus physiological constraints alone may only partially explain the restriction of *A. blancae* breeding to the first rainy season (Martin 2004). Unfortunately, data on molt and other physiological processes are lacking for this species.

We also found that photoperiod was associated with breeding activity, even after accounting for its collinearity with precipitation. Photoperiod is a well‐established cue in temperate birds (Lack 1968; Dawson et al. 2001). As days lengthen, birds increase reproductive activity for ecological and physiological reasons (Dawson and Sharp 2007; Wingfield and Farner 1993). In tropical species, photoperiod has also been shown to influence breeding timing (Hau et al. 1998, 2008; Wikelski et al. 2000; Castaño et al. 2023), though generally less strongly than in temperate regions. It has been hypothesized that tropical species farther from the equator (10°–20° latitude) rely more on photoperiod as a reproductive cue (Hau 2001; Wikelski et al. 2000). Although day length varies less in the tropics, it remains more predictable than other environmental cues and may serve as an indirect signal of forthcoming resource pulses. This may explain the correlation we observed between breeding and photoperiod in *A. blancae* . Indeed, studies of opportunistic breeders show that reproduction can be triggered by increasing food availability during longer days, suggesting that photoperiod may act primarily as an indirect trigger of breeding activity (Perfito et al. 2008).

Finally, it is possible that El Niño Southern Oscillation (ENSO) events influenced the timing of reproduction during the time of our study. In Colombia, 2021 and 2023 were years influenced by La Niña, in which more rain was received than the average. Traditionally an excess of rain may result in an earlier or an additional resource pulse that could trigger reproduction during these years. Nonetheless, in our study area, data provided by meteorological stations suggested that neither 2022 nor 2023 were abnormally wet suggesting that our sampling was not influenced by ENSO events.

### 5.2 Morphology

We found significant morphological differences between sexes and across life stages. Males were generally larger than females, and these differences were more pronounced in subadults than in adults. This suggests that multiple mechanisms may act independently on males and females (Caron and Pie 2024). Sexual size dimorphism is often attributed to sexual selection, since larger males are better able to defend larger or more productive territories (Owens and Hartley 1998; Caron and Pie 2024). Alternatively, niche differentiation or phylogenetic history may also explain the observed patterns (Shine 1989). With the current data, we cannot distinguish among these hypotheses, but it is notable that such sexual size dimorphism is uncommon within this genus and even within the family.

Furthermore, we provide a dataset of 29 females and 25 males that can be used to approximate sex using noninvasive techniques, supporting demographic monitoring (Ellrich et al. 2010). Wing length and total length showed the strongest differences between sexes. On average, males had a wing length of 74.7 mm, whereas females averaged 70.1 mm. Notably, interquartile ranges did not overlap: males ranged from 74 to 76 mm, while females ranged from 69 to 71 mm. Females were also nearly 5 mm shorter in total length (mean = 156.8 mm, IQR = 152–160 mm) compared with males (mean = 161.2 mm, IQR = 158–165 mm), though here the ranges slightly overlapped. Thus, in field conditions, individuals with a wing length of 74–76 mm and a total length of 158–165 mm are very likely to be males.

Previous studies have noted differences in plumage coloration among age classes of *A. blancae* (Correa et al. 2019; Chaparro‐Herrera and Lopera‐Salazar 2019). Here, we show that in addition to coloration, there are also clear morphological differences. Adult males were the largest individuals, mainly due to longer tails. Gape, tail length, and total length were particularly useful for distinguishing life stages. Juveniles tended to have larger gapes and shorter tails, resulting in a smaller total length, consistent with their recent fledging and incomplete plumage. Nonetheless, coloration patterns—especially gape coloration—remain the most reliable traits for distinguishing age classes, with juveniles showing more bulging, brightly colored gapes compared to adults (Ralph et al. 1996). We propose, however, that the measurements in our dataset may serve as a complementary classification key, particularly in cases where plumage‐based identification is ambiguous. Accurate classification of life stages is crucial for parameterizing population viability models, which in turn can inform conservation strategies targeting different life stages.

There are some examples in passerine birds in which males increase size during the breeding season due to increases in the size of reproductive organs, muscle mass or fat storage and this dimorphism is not maintained year‐round (Meijer et al. 1994). The increase in size has been attributed to competition between males for territories. Alternatively, females may reduce their mass during incubation (Meijer et al. 1994). In any case, there are few reports of other morphological traits showing seasonal size differences between sexes (see Greenberg et al. 2013). Consequently, we stress however that further research should focus on determining whether adult sexual differences occur only during breeding or persist across life‐history stages.

## 6 Conclusions

By documenting breeding phenology and providing quantitative morphological measurements, this study advances knowledge of *A. blancae* and represents an essential step toward situating this species within the broader context of avian reproductive biology. Although our study significantly expands knowledge of the natural history of *A. blancae* and provides data relevant to conservation (Hampton and Wheeler 2012), further work is needed. More detailed data on nesting times later in the calendar year would clarify whether the species shows one or two reproductive peaks. Combining such data with information on subadult and juvenile survival will improve understanding of population trends across its restricted range in the Altiplano of Santa Rosa de Osos.

## Author Contributions

**Juan Pablo Gomez:** conceptualization (equal), data curation (equal), formal analysis (equal), funding acquisition (equal), investigation (equal), methodology (equal), project administration (equal), resources (equal), validation (equal), visualization (equal), writing – original draft (equal), writing – review and editing (equal). **Sergio Chaparro‐Herrera:** conceptualization (equal), data curation (equal), formal analysis (equal), funding acquisition (equal), investigation (equal), methodology (equal), project administration (equal), resources (equal), validation (equal), visualization (equal), writing – original draft (equal), writing – review and editing (equal).

## Conflicts of Interest

The authors declare no conflicts of interest.

## Supporting information


**Data S1:** ece372408‐sup‐0001‐SupinfoS1.csv.

**Data S2:** ece372408‐sup‐0002‐SupinfoS2.csv.

**Data S3:** ece372408‐sup‐0003‐SupinfoS3.docx.

## Data Availability

All data used in this study has been uploaded as Supporting Information [Link], [Link], [Link].
