# Supplementary material for: Seasonal Breeding and Morphological Variation Across Age and Sex in the Antioquia Brushfinch (Atlapetes blancae)
Source: Ecol Evol. 2025 Nov 12;15(11):e72408. doi: 10.1002/ece3.72408 (PMC12611420; doi:10.1002/ece3.72408)

**Supplementary Information**

We provide the raw data used for all analyses in this study as supplementary information. The **PhenologicalDataset.csv** contains the presence absence data for all evidence, juvenile only and other only evidence for each week in the year. Additionally, it contains data about mean weekly precipitation and day length to conduct regression analyses to evaluate the effect of precipitation and day length on the probability of reproduction. The file **MorphologicalDataset.csv** file contains the information of morphological measurements for all 55 individuals captured along with data about its sex and age.

**Details of dates and locations of individuals captured for morphological analyses.**

Of the total number of individuals captured, six were juveniles, three were captured between May 16 and 23, 2022 and two between July 14 and 16, 2022 in Aragon, and one in San Pedro on June 15, 2022. Twelve immature individuals were also captured, seven in Aragon, one on May 23, 2022, six between July 13 and 16, 2022, and five in San Pedro between June 2 and 16, 2022. Finally, between May 17 and 25, 2022 in Aragón, seven individuals with incubation patches were captured, and three between July 13 and 16, 2022; an individual with an incubation patch was also captured on June 2, 2022, in San Pedro. In turn, 16 individuals with a cloacal protrusion were captured between May 16 and July 16, 2022, 15 in Aragon and one in San Pedro.

**Supplementary Tables**

**Table S1.**

Loadings and eigenvalues resulting from the principal components analysis performed on morphological traits of Antioquia Brushfinch (*Atlapetes blancae*). In bold, we show the largest loading to the principal component.

|  | **PC1** | **PC2** | **PC3** | **PC4** |
| --- | --- | --- | --- | --- |
| Weight | **0.33** | 0.24 | -0.29 | 0.13 |
| Bill Height | 0.25 | -0.04 | **0.39** | 0.38 |
| Bill Width | -0.04 | -0.01 | **0.48** | -0.32 |
| Gape | -0.20 | -0.10 | 0.08 | **0.73** |
| Total Culmen | 0.04 | **-0.68** | -0.03 | -0.02 |
| Exposed Culmen | 0.29 | **-0.52** | 0.17 | 0.11 |
| Tarsus | 0.08 | **0.43** | 0.36 | 0.28 |
| Hallux | -0.15 | -0.02 | **0.49** | -0.10 |
| Wing Length | **0.50** | -0.01 | 0.02 | 0.10 |
| Tail Length | **0.43** | 0.10 | 0.29 | -0.29 |
| Total Length | **0.48** | 0.03 | -0.22 | 0.01 |
| Eigen values | 1.57 | 1.34 | 1.25 | 1.08 |
| Prop. Variance | 0.22 | 0.16 | 0.14 | 0.11 |

**Supplementary Figures**

**Figure S1**.

Biplot resulting from the principal components analysis performed on morphological traits of Antioquia Brushfinch (*Atlapetes blancae*). We only show the loadings of the variables most related to each principal component.


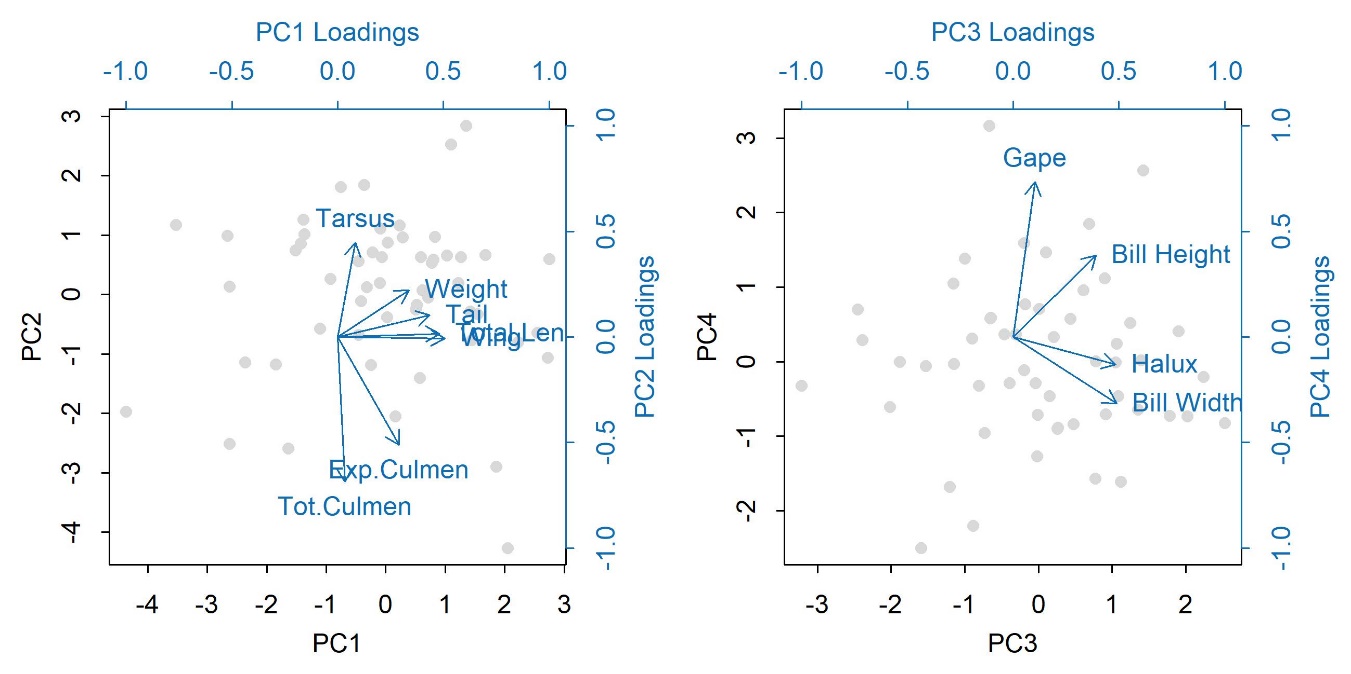


**Figure S2**. Boxplots showing the comparisons of raw morphological traits between individuals in different age stages and sex of Antioquia Brushfinch (*Atlapetes blancae*).


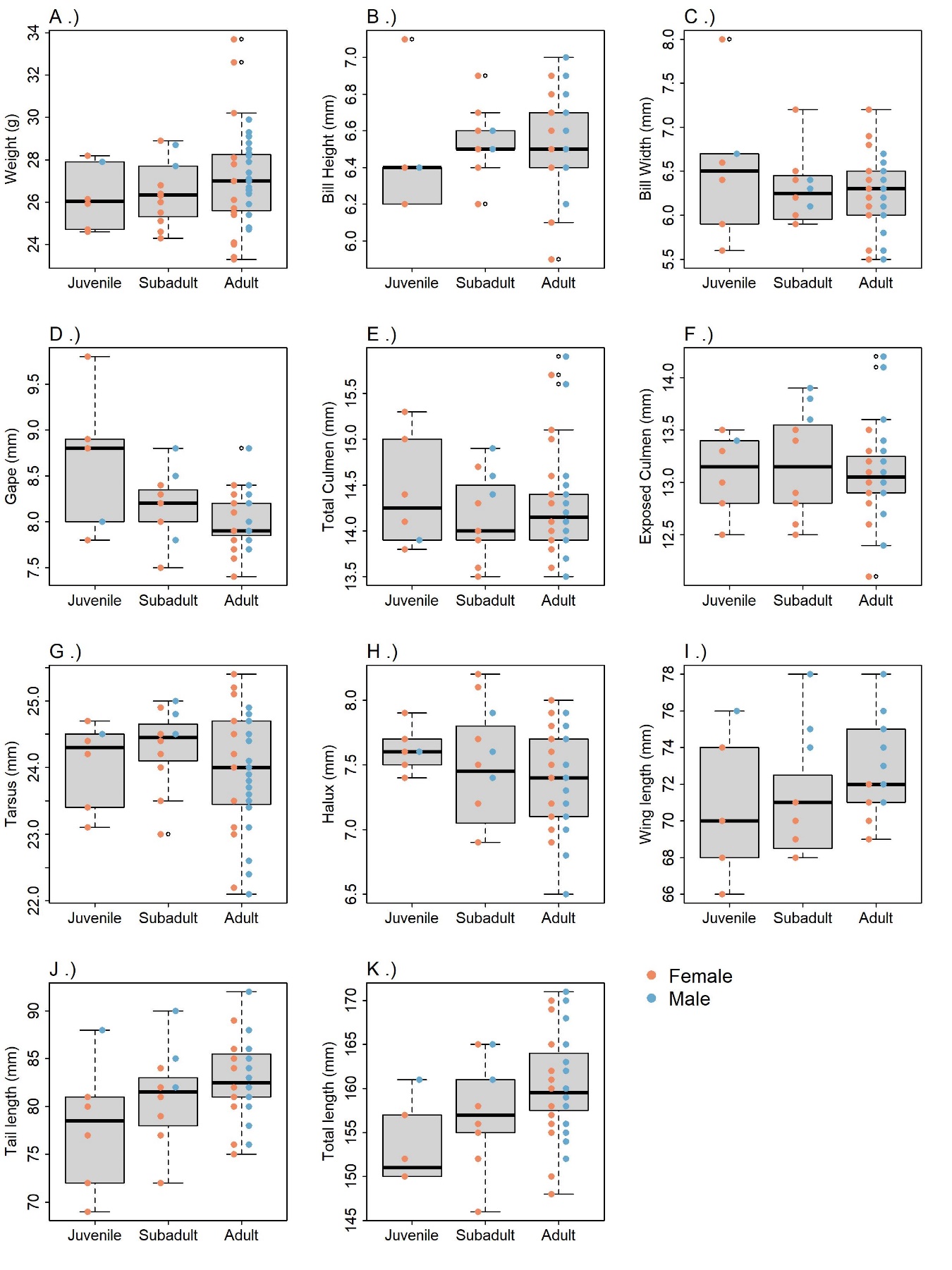

Supplement: Supplementary file 3 — Data S3: ece372408‐sup‐0003‐SupinfoS3.docx. [file ECE3-15-e72408-s003.docx]
